# Progesterone-induced changes in the phosphoryl potential during the meiotic divisions in amphibian oocytes: Role of Na/K-ATPase

**DOI:** 10.1186/1471-213X-11-67

**Published:** 2011-11-06

**Authors:** Gene A Morrill, Terry L Dowd, Adele B Kostellow, Raj K Gupta

**Affiliations:** 1 Department of Physiology and Biophysics, Albert Einstein College of Medicine, Bronx, New York 10461, USA; 2 Department of Chemistry, Brooklyn College of the City University of New York Brooklyn, New York 11210, USA

**Keywords:** ^31^P-NMR, phosvitin, Na/K-ATPase, meiosis, oocytes, bioenergetics

## Abstract

**Background:**

Progesterone triggers resumption of the first meiotic division in the *Rana pipiens *oocyte by binding to the N-terminal external loop of the catalytic subunit of Na/K-ATPase, releasing a cascade of lipid second messengers. This is followed by internalization of specific membrane proteins, plasma membrane depolarization and nuclear membrane breakdown, culminating in arrest at second metaphase.

**Results:**

Progesterone initiates an increase in phosphoryl potential during the first meiotic division, resulting in the accumulation of high energy protein phosphate by second metaphase arrest. ^31^P-NMR, with saturation transfer, demonstrates that the phosphocreatine level rises ~2 fold and that the "pseudo" first order rate constant for the creatine kinase reaction falls to ~20% of the control by the onset of nuclear membrane breakdown. ^32^PO_4 _pulse-labeling reveals a net increase in phosphorylation of yolk protein phosvitin during this period. The increased yolk protein phosphorylation coincides with internalization of membrane Na/K-ATPase and membrane depolarizatio

**Conclusions:**

These results indicate that progesterone binding to the catalytic subunit of the Na-pump diverts ATP from cation regulation at the plasma membrane to storage of high energy phosphate in yolk protein. Phosvitin serves as a major energy source during fertilization and early cleavage stages and is also a storage site for cations (e.g. Na^+^, K^+^, Ca^2+^, Fe^2+/3+^) essential for embryonic development.

## Background

The amphibian ovarian oocyte is blocked in first meiotic prophase until a transient rise in gonadotropin stimulates its surrounding follicle cells to release progesterone [1], which binds to the first external loop of the catalytic subunit of the Na//K-ATPase at the oocyte surface to reinitiate the meiotic divisions [2,3]. The oocytes then complete one and one-half meiotic divisions, are released from the ovarian follicle, and become blocked at second meiotic metaphase. Sperm penetration results in the completion of meiosis followed by a period of rapid mitoses characteristic of the developing blastula. *Rana pipiens *ovaries contain a single population of mature oocytes that respond to gonadotropin, in contrast to the multiple growth stages seen in *Xenopus laevis *when maintained under laboratory conditions [4].

Our previous studies with *Rana pipiens *oocytes showed that, *in vivo*, gonadotropin induces phosphorylation of the yolk protein, phosvitin, and that the release of the metaphase block by fertilization and the subsequent synchronous cell divisions coincide with stepwise phosvitin dephosphorylation [5]. In the present study, we have analyzed changes and turnover in high energy phosphates during the meiotic divisions using ^31^P NMR and ^32^PO_4 _pulse labeling techniques *in vitro*. *Rana pipiens *oocytes are excellent experimental material for noninvasive NMR studies of cell division because of their large size and the ease of superfusion in an NMR tube, which maintains physiological oxygen levels. Oocytes from each female undergo synchronous meiotic divisions. The prophase-arrested (control) oocytes maintain a sizeable pool of high energy phosphate compounds, including phosphocreatine (PCr), ATP and serine-rich phosphoproteins, for at least 24 h during superfusion [6].

Little has been published about the compartmentation or turnover of high energy phosphates within oocytes or the bioenergetic changes during meiotic division. Using ^31^P-NMR and the saturation transfer technique, we have examined the effect of the physiological meiotic inducer (progesterone) on both the forward PCr → ATP and the reverse ATP → PCr rates of the creatine kinase reaction in *Rana *oocytes during the first meiotic division. The ^31^P-NMR measurements were correlated with *in vitro *^32^PO_4 _turnover using pulse labeling techniques in oocytes (free of epithelial cells) undergoing synchronous meiotic divisions.

We find that *in vitro *progesterone initiates an increase in phosphoryl potential and that phosphorylation of the yolk protein phosvitin is accompanied by internalization of the ouabain-sensitive Na/K-ATPase and plasma membrane depolarization. This indicates that a progesterone-induced shift in high energy phosphate utilization from cation pump to phosvitin phosphorylation is necessary for completing the meiotic divisions and early development.

## Results

### Intracellular environment of the prophase *Rana pipiens *oocyte

**Figure 1:**
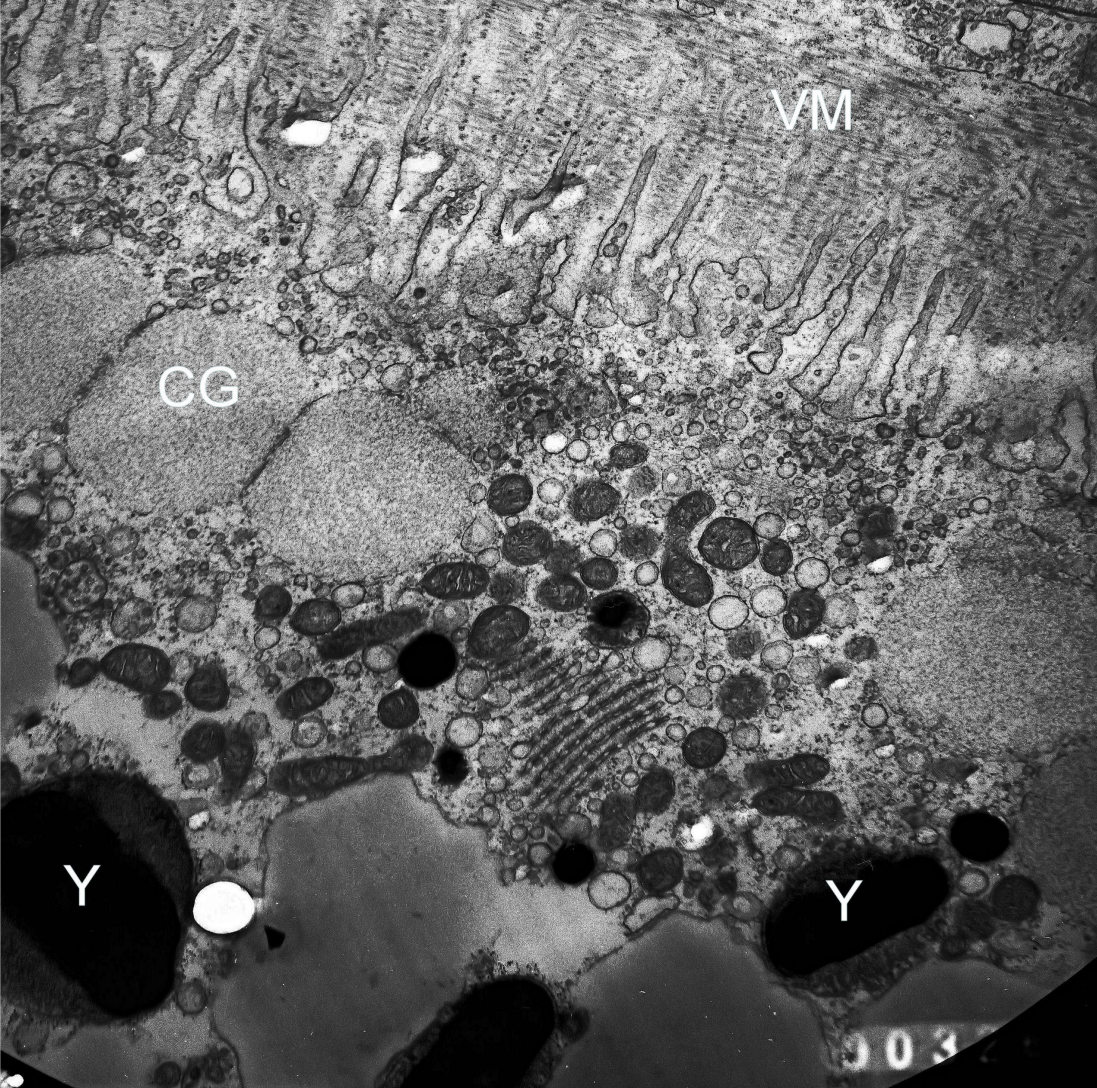
**A transmission electron micrograph (×12,000) of a prophase-arrested, untreated ovarian follicle from hibernating *Rana pipiens***. An area of the oocyte cortex with vitelline membrane (VM), oocyte surface microvilli, cortical granules (CG) and yolk platelets (Y) was selected. Annulate lamellae (membrane array below center of figure), numerous mitochondria and other vesicles are visible. Follicles were fixed sequentially with OsO_4 _and glutaraldehyde and post-fixed in 1% buffered OsO_4 _for 1.5 h as described [18]. Samples were embedded in Epon and 50 to 80 nm sections were stained with uranyl acetate and then counter stained with lead citrate. Micrographs were taken using a Jeol 100 CX electron microscope at 80 KV.

Figure 1 illustrates a transmission electron micrograph (× 12,500) of the fully grown *Rana pipiens *oocyte in prophase arrest. The micrograph depicts a cortical region of the animal hemisphere of an unstimulated prophase *Rana pipiens *oocyte showing the interface between the membrane microvilli and the inert matrix, called the vitelline membrane (VM), on the oocyte surface. Two of the large yolk platelets are indicated (Y) and three large cortical granules (CG) are visible at the microvillar interface. Numerous small round mitochondria can be seen just below the oocyte plasma membrane. The system of stacked membranes below center are the annulate lamellae [7]. In contrast to the animal hemisphere, the vegetal hemisphere is packed with yolk platelets of varying sizes (not shown)

The ultrastructure of yolk platelets from *Rana *oocytes consists of a central main body with a crystalline lattice structure, with an enclosing membrane approximately 70 **Å **in thickness [8]. Electron micrographs of the main body reveal hexagonal net, square net and parallel band patterns. The lipoprotein complex, vitellogen, contains 12% lipid and smaller amounts of carbohydrates and biliverdin [9]. Fluorescence confocal microscopy, using a pH-sensitive fluorescent dye, indicates that the mature yolk platelets are acidic (pH 5.6) [10]. As shown previously, about 85% of the *Rana pipiens *oocyte dry weight is recovered with the yolk platelet fraction isolated by differential centrifugation of 0.24 M sucrose homogenates [11].

### ^31^P-NMR analysis of high energy phosphate turnover

**Figure 2:**
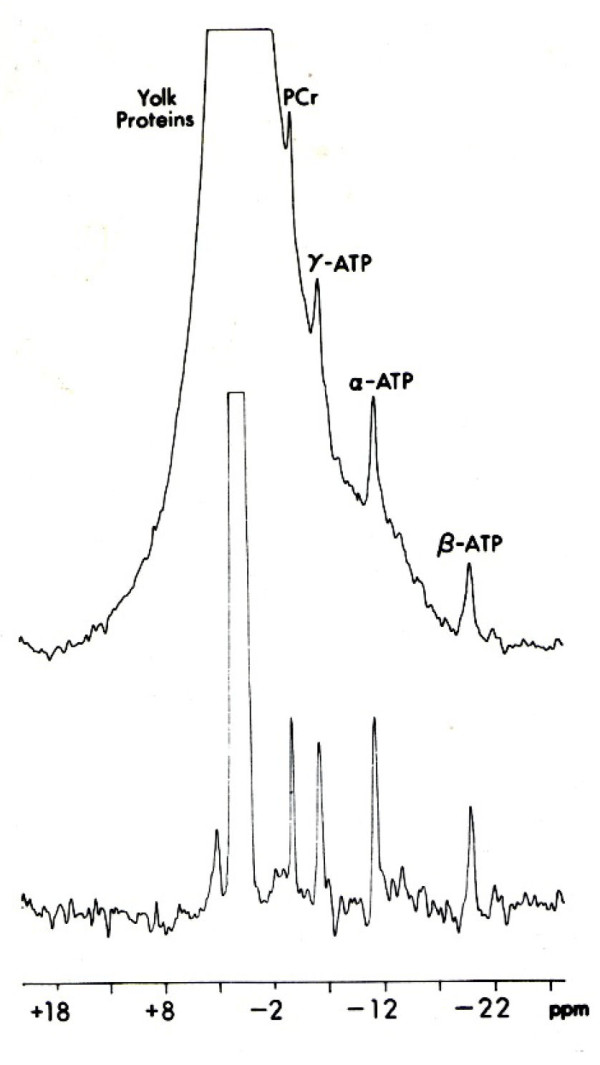
**^31^P-NMR spectra of 200-250 *Rana pipiens *follicles in meiotic prophase-arrest obtained with (lower) and without (upper) the use of convolution difference to minimize the broad phosphoprotein signal**. Sampling time was 40 min; follicles were superfused with Ringer's solution at 1 ml/min as illustrated in Figure 8.

The ^31^P-NMR spectrum of *Rana pipiens *follicles is shown in Figure 2. The outstanding feature of the ^31^P-NMR spectra of frog oocytes is the resonance of the yolk phosphoprotein, with additional resonances for phosphocreatine (PCr) and the α, β and γ phosphoryl groups of ATP (upper spectrum, Figure 2). The resonance of the intracellular inorganic phosphate is buried under the large yolk phosphoprotein signal and is totally unobservable, even in the form of a shoulder riding on the large peak. Use of the convolution difference technique alone (lower spectrum, Figure 2) is useful for resolving creatine phosphate, and the α, β, and γ resonances of ATP, although insufficient to resolve the P_i _peak. Techniques based on differential relaxation properties of the narrow P_i _resonance have been used for observing and defining the position of the intracellular P_i _resonance [6]. Saturation transfer experiments were carried out by application of a low power RF pulse for a time of 3T_1 _to either the γATP or PCr resonance. Control spectra were obtained by positioning the saturating pulse symmetrically on the other side of PCr or γATP, respectively (see Methods).

**Figure 3:**
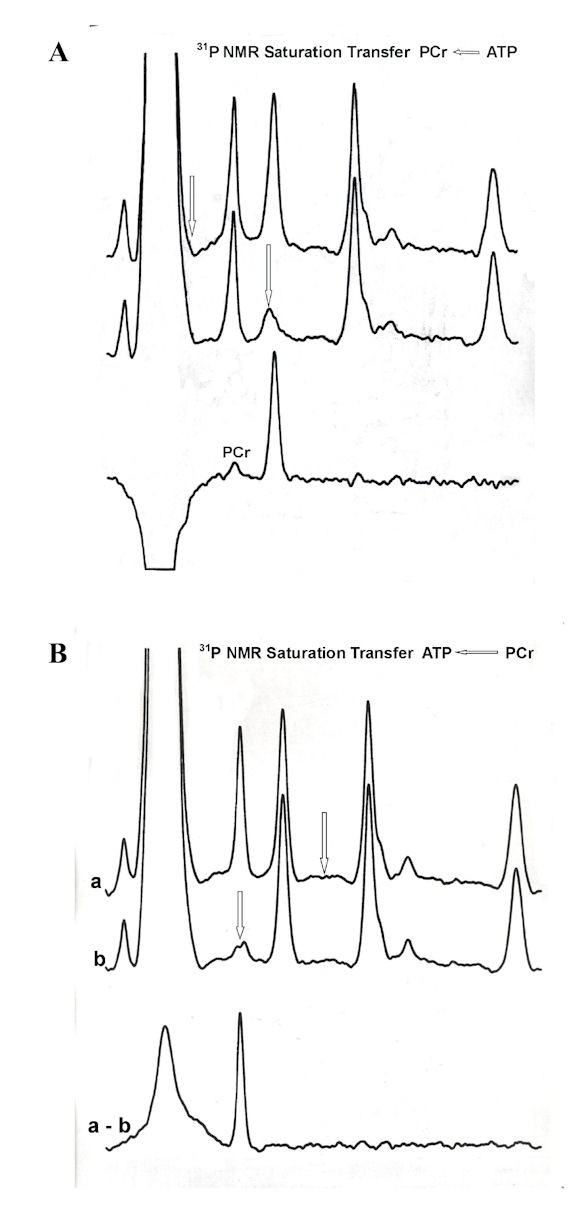
**^31^P saturation transfer NMR spectra of untreated *Rana Pipiens *follicles**. Arrows indicate where saturation was alternately applied. **Upper spectra (A)**: ^31^P NMR showing saturation transfer from γP → PCr. The control spectrum with saturating RF placed off-resonance (upper trace), the spectrum with γP resonance saturated (middle trace) and the difference spectrum (lower trace) are compared. **Lower spectra (B)**: ^31^P NMR saturation transfer results for PCr → γP. The control spectrum (a), the spectrum with PCr saturated (b), and the difference spectrum (a - b). Each spectrum was obtained with a selective low power RF pulse of 5 sec duration placed in the labeled (arrow) position, followed by a 90° nonselective observation pulse and a 0.8 sec acquisition time. A total of 1000 transients were collected at 20°C for each spectrum by alternating the saturating RF between an off resonance and the γP (or PCr) peak positions after each block of 100 transients, as described in the text. A line-broadening of 100 Hz was applied to each spectrum. The large peaks in the difference spectra are due to incomplete cancellation of the overwhelming phosvitin signal.

The difference spectrum showing transfer of saturation from γATP to PCr resonance is illustrated in the upper panel (A) of Figure 3, whereas that for reverse transfer from PCr to γATP is shown in the lower panel. After saturation of the γATP resonance there was a 19% transfer of saturation to the PCr resonance. The T_1 _of PCr resonance measured in the presence of the saturation of γATP was 2.16 ± 0.09 sec which, using theoretical formulation [12], yielded a pseudo first order rate constant (k_f_) for the PCr → ATP reaction of 0.09 ± 0.01 sec^-1^. The PCr concentration of the prophase oocyte was measured to be 2.04 ± 0.31 mM (N = 3) giving a forward flux of 0.18 ± 0.03 mM/sec. However, when PCr was saturated, there was no detectable transfer of saturation to γATP in the difference spectrum. This may reflect compartmentation of ATP in non-cytosolic compartments [13,14].

**Figure 4:**
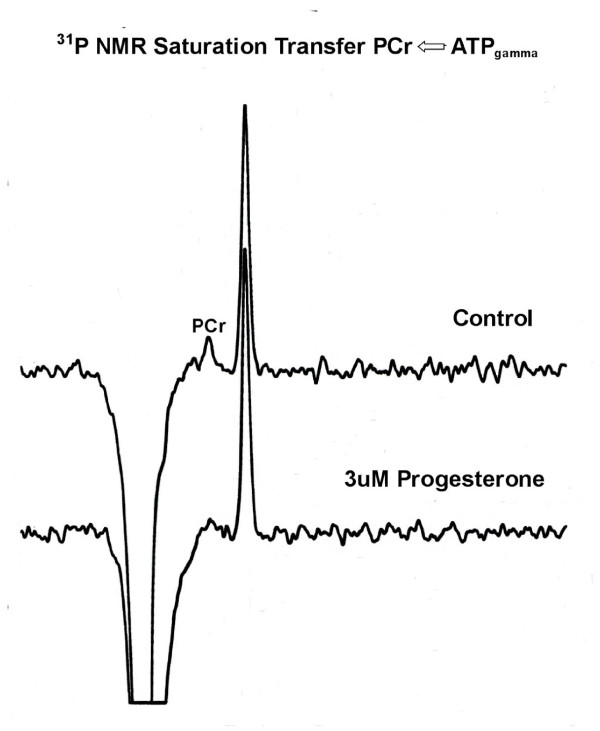
**A comparison of the saturation transfer difference spectra of follicles superfused with Ringer's solution (control, upper spectrum) and Ringer's solution containing inducing levels (3.2 UM) of progesterone (lower spectrum) at 20-22°C**. The midpoint of the spectral data aquisition corresponded to a 5.5 h exposure to progesterone. A marked decrease in the γP → PCr saturation transfer effect is observed in progesterone-treated oocytes. Again, the large inverted peaks are due to incomplete cancellation of the overwhelming phosvitin signal.

**Figure 5:**
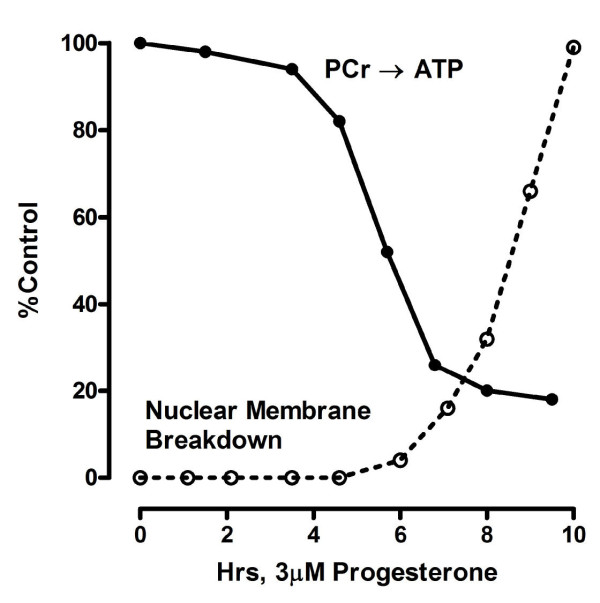
**Comparison of changes in the NMR-measured pseudo first order rate constant (k_f_) for the reaction PCr → ATP and the time course of nuclear membrane breakdown during the first 10 h**. Values are expressed as a percent of those for untreated follicles from the same female.

Figure 4 compares the difference spectra of follicles superfused with Ringer's solution (control, upper spectrum) and with Ringer's solution containing inducing levels of progesterone (3.2 μM progesterone, lower spectrum) at 20-22°C. A 14 ± 2% (N = 3) increase in PCr was seen after a 3 h incubation in progesterone with a 79 ± 4% increase after 10 h. The extent of the saturation transferred to PCr from ATP is, however, significantly reduced in the presence of progesterone, as indicated by the loss of intensity of the PCr resonance in the difference spectrum (lower trace). Figure 5 compares the time course for the change in the pseudo first order rate constant for the PCr → ATP reaction with % nuclear membrane breakdown over the first 11 h in progesterone-treated follicles from the same female. As shown, the NMR-measured rate constant began to decrease after about 3-4 h of continuous exposure to steroid, and approached about 15% of untreated oocytes by 10 h. Nuclear membrane breakdown began as the rate constant approached a minimum value. After 10 h, the pseudo rate constant was 17 ± 3% (N = 3) in progesterone-treated follicles compared to the control. No measurable changes in PCr levels or in the rate constant were seen in control follicles superfused with modified Ringer's solution for up to 24 h. (Unlike *Xenopus laevis*, nuclear membrane breakdown is never seen in control (untreated) *Rana pipiens *follicles.)

### Progesterone stimulation of protein phosphorylation in *Rana *oocytes

**Figure 6:**
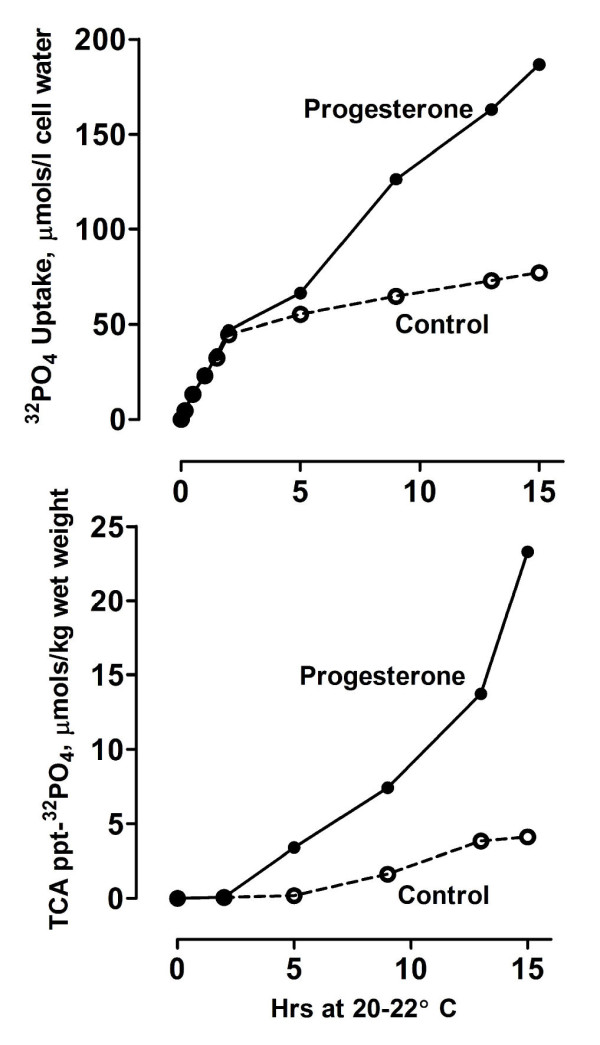
**Comparison of ^32^PO_4 _uptake by control and progesterone-treated *Rana pipiens *ovarian follicles**. **Upper panel**: *in-vitro *[^32^PO_4_] uptake by control and progesterone-treated denuded oocytes. Oocytes were incubated in Ringers' solution containing 0.08 mM NaHPO_4 _and ^32^PO_4 _uptake is expressed as μmols/1 cell water. **Lower panel**: oocytes from the upper panel were homogenized in 7% TCA and protein isolated as described in Methods. ^32^PO_4 _uptake into total protein is expressed as μmols/kg wet weight.

The upper panel of Figure 6 compares ^32^PO_4 _uptake from the medium by control and progesterone-treated *Rana *ovarian follicles, expressed as moles/liter of oocyte water. Extracellular inorganic PO_4 _levels were maintained at 80 μM. Uptake was essentially identical in control and progesterone-treated oocytes during the first 5 h, but markedly increased in progesterone-treated oocytes prior to the onset of nuclear membrane breakdown, continuing to rise even after completion of the membrane dissolution. The lower panel of Figure 6 compares ^32^PO_4 _incorporation into the TCA precipitable components of the control and progesterone-treated oocytes shown in the upper panel. In progesterone-treated oocytes, 13 ± 2% (N = 3) of the total ^32^PO_4 _taken up is incorporated into total protein, phospholipid and nucleic acid components by completion of nuclear membrane breakdown, compared to 5 ± 1% (N = 3) incorporation in control oocytes over the same time period.

Since the major increase in protein phosphorylation occurred after 4 - 6 h exposure to inducing levels of progesterone (Figure 6), isolated follicles were preincubated in Ringer's solution containing 3.2 μM progesterone for 5 h, then pulse labeled with Ringer's solution containing ^32^PO_4 _and 3.2 μM progesterone for 4 h. The oocytes were rinsed, homogenized at ice-bath temperatures and phosvitin isolated as described in Methods. Table 1 compares ^32^PO_4 _uptake into purified phosvitin in control and progesterone-stimulated follicles. A 15 fold increase in ^32^PO_4 _incorporation was observed in phosvitin isolated from progesterone-treated follicles. Compared to the controls, 3.2 μM progesterone produced a 22 ± 4% (N = 3) increase in phosvitin phosphate, based on phosphate analysis [5].

**Table 1:** Comparison of [^32^PO_4_] Incorporation into Purified Phosvitin from Control and Progesterone-treated *Rana pipiens *Follicles^a^

Protein Fraction	Control	Progesterone
	Sp. Act. Cpm/ìmole protein phosphate^b^	
Purified Phosvitin	160 ± 36	2368 ± 104

^a^Isolated ovarian follicles were preincubated in Ringer's solution with or without 3.2 jaU progesterone for 5 h at 22°C, [^32^PO_4_] added, and then further incubated for 4 h. ^b^Mean ± SD (N = 3) Denuded oocytes and/or isolated ovarian follicles from 3 hibernating *Rana pipiens *females.

### Structure of phosvitin in amphibians

**Figure 7:**
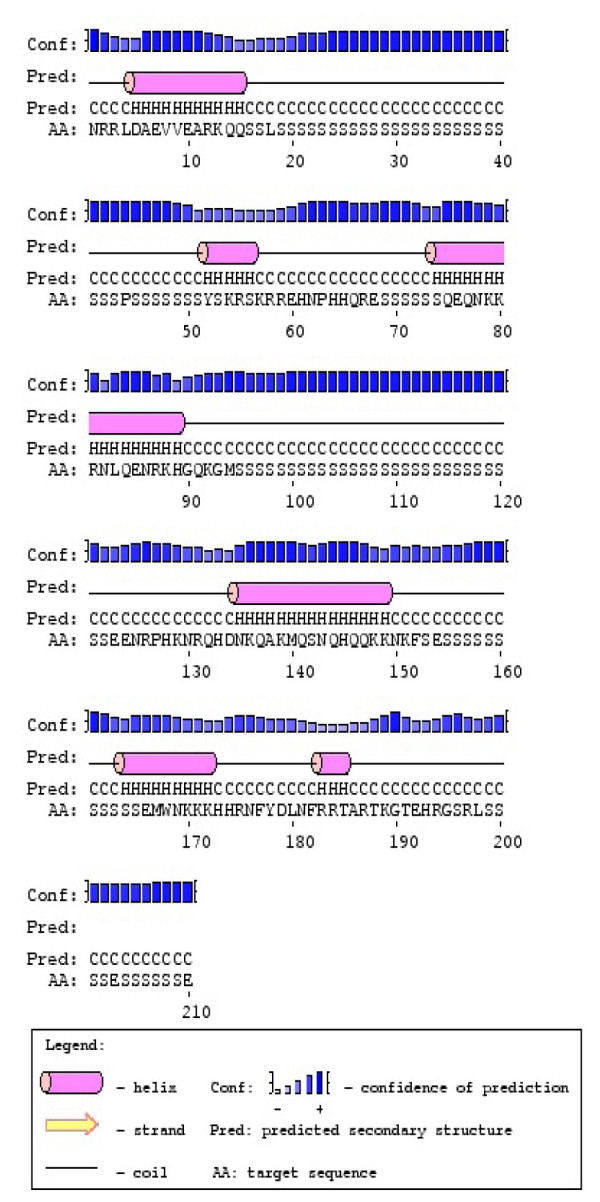
**Predicted structure of the frog phosvitin component of Vitellogenin-A2 (1807 amino acids; Accession #P18709)**. The Protein Structure Prediction Server (PSIPRED) [16] was used to examine the possible conformation of the serine-rich phosvitin sequence (ca. 1126 - 1321). Line 1 indicates the confidence of the prediction, line 2 the relative position of each helix, line 3 whether the residue is part of a helix, strand or coil (see legend), and line 4 indicates the amino acid. Going from N- to C-terminal end, the sequence contains blocks of 38, 8, 28, 13 and 13 serine residues.

Phosvitin is the principal phosphoprotein in the eggs of numerous vertebrates (reviewed in [15]). In *Xenopus laevis*, a species closely related to *Rana pipiens*, phosvitin is one of three chains in the protein complex called Vitellogenin-A2 (1807 amino acids; Accession #P18709). The Protein Structure Prediction Server (PSIPRED) [16] was used to examine the secondary structure of the serine-rich phosvitin sequence (ca. 1126 - 1321). As shown in Figure 7, the predicted structure was relatively complex, with 6 α-helixes. Going from the N- to C-terminal ends, the sequence contains blocks of 38, 8, 28, 13 and 13 serine residues. Serines are generally excluded from helix-containing regions. Thus, each phosvitin molecule contains at least 100 serine residues as possible phosphorylation-dephosphorylation sites. Based on our data, it appears that progesterone selectively stimulates phosphorylation of about one of every 5 serines.

## Discussion

The results presented here and elsewhere [2,3] indicate that progesterone binding to the catalytic subunit of the Na/K-ATPase at the oocyte plasma membrane initiates a sequence of changes in high energy phosphate compounds during the first meiotic division. In *Rana pipiens *oocytes, breakdown of the nuclear membrane occurs 8-10 hours after exposure to inducing levels of progesterone, followed by arrest at second meiotic metaphase at 15-16 hours (reviewed in [17]). An 80% increase in PCr precedes nuclear membrane breakdown accompanied by a marked decrease in pseudo first order rate constant (k_f_) for the PCr → ATP reaction (Figure 5). This increase in phosphoryl potential coincides with an increased phosphorylation of the phosphate-rich yolk protein, phosvitin, beginning at the onset of nuclear membrane breakdown (Figure 6).

### Changes in ATP utilization and plasma membrane surface area during the meiotic divisions

The decrease in k_f _shown in Figure 4 indicates a drop in cytosolic ADP concentration and an increase in the cytosolic phosphoryl potential of the oocyte prior to nuclear membrane breakdown (4 - 6 h). The decreased availability of ADP, normally generated in large part by hydrolysis of ATP during ion-transport by Na/K-ATPase, could arise from the observed internalization of the plasma membrane Na/K-ATPase (Na-pump) over the same time period [2]. This seems probable, based on our data showing a simultaneous 50 - 60% decrease in membrane capacitance (cell surface area), increased endocytosis [18], and the disappearance of more than 95% of the high affinity [^3^H]ouabain binding sites in progesterone-treated denuded *Rana pipiens *oocytes over the same time period [2].

[^3^H]ouabain specifically binds to the α-subunit of the Na/K-ATPase (reviewed in [2,3]). By 6 h the [^3^H]ouabain is recovered bound to intracellular vesicles of progesterone-treated oocytes; it does not diffuse out of the progesterone-treated denuded oocytes [2]. Both scanning [18] and transmission (Figure 1) electron micrographs indicate that the prophase oocyte is covered with numerous microvilli. Estimates of the surface area in control oocytes reveal that the microvilli increase the oocyte surface area 10-12 times compared with that of a sphere of the same diameter [18]. Scanning electron micrographs reveal that the microvilli of progesterone-treated oocytes disappear coincident with the decrease in membrane capacitance, leaving only stumps on the oocyte surface [18].

### Phosphorylation of yolk platelet phosvitin

As seen in the ^31^P-NMR spectrum in Figure 2, phosvitin is the major phosphoprotein in the amphibian oocyte. Phosvitin is a glycosylated, serine-rich peptide with reported masses of 16-19 kDa, 25 kDa, or 31 kDa (reviewed in [19]). A single resonance (2.59 ppm) dominates the proton-decoupled ^31^P-NMR spectrum of *Xenopus *phosvitin [20]. Comparison of the phosvitin spectra with and without proton decoupling suggests a triplet splitting pattern for the major resonance, presumably due to coupling to methylene protons.

Rabinowitz and Lipmann [21] were the first to demonstrate reversible phosphate transfer between yolk phosphoprotein and ATP. Attempts were made to determine the equilibrium constant of the reaction between ATP and phosphoprotein. Figures varying from 20 to 50 were obtained for the forward reaction. However, their experiments indicated a non-homogenous phosphate population. The authors suggested that the "thermodynamic potential of phosphoryl (groups) in phosvitin to be not far below that of ATP". Mano and Lipmann [22] subsequently found that only more highly phosphorylated forms of phosvitin were good acceptors of phosphate from protein kinase and ATP. This suggests that a large fraction of the phosvitin serine phosphates do not turn over *in situ*. Our data indicate (Table 1) that only a small fraction of the serine phosphates in yolk phosvitin may be available for reversible phosphoryl exchange with ADP/ATP.

Phosvitin also contains firmly bound, non-heme iron [23]. Grant and Taborsky [24] suggested that at alkaline pH, autoxidation of iron converts phosvitin-bound serine phosphate to the corresponding enol phosphate, an energy-rich structure. However, subsequent studies by Rosenstein and Taborsky [25] failed to find evidence for the production of a stable phosphoenol product and a demonstration of the stability of the C-H bond at the α-carbon of the oxidized residue further ruled it out. Their finding that phosphate release occurs by P-O bond cleavage is consistent with a mechanism by which an oxidatively generated carbonium ion derivative of phosphoserine is converted into a stable product by the direct formation of the free aldehyde and a monomeric metaphosphate ion, the latter reacting with water to yield inorganic orthophosphate. Rosenstein and Taborsky [25] proposed that yolk phosvitin would provide the developing embryo with a potential phosphorylating agent (HPO_4_^2-^) which becomes activated by oxidation.

### Progesterone-induced protein phosphorylation

Phosvitin phosphate turnover is minimal in control oocytes (Table 1) and the 15-fold increase in ^32^PO_4 _incorporation in progesterone-treated oocytes reflects the increased phosphate turnover in one phosphate per block of 5 serine phosphates in hormone stimulated oocytes, indicating that specific serine residues of highly phosphorylated species of yolk phosvitin are further phosphorylated in response to progesterone. This is consistent with the proposal by Williams and Sanger [26] that structures containing serine-phosphoserine blocks could serve as active sites in cellular metabolism.

The failure to observe a progesterone-induced increase in ^32^P-labelling of phosvitin in *Xenopus laevis *ovarian follicles by Maller et al. [27] may be due, in part, to dephosphorylation by endogenous ferrous/ferric ions during protein isolation. Additionally, *Xenopus laevis *ovaries contain only a small subpopulation of "banded" oocytes that are selectively released by gonadotropin [4]. Thus, increased phosvitin phosphorylation may only occur in a small subpopulation of *Xenopus laevis *oocytes that are exposed to progesterone. In comparison, 100% of the large *Rana pipiens *oocytes respond to, and are released from, the follicles by gonadotropin. The *Xenopus *phosphorylation studies should be repeated using the "banded" oocyte population.

Taken *in toto*, our findings indicate that progesterone initiates a selective internalization of plasma membrane rich in Na/K-ATPase (Na-pump sites). This decrease in Na/K-pump activity coincides with plasma membrane depolarization and significantly reduces the ATP utilized for ion transport. Increased phosphorylation of the yolk protein phosvitin coincides with membrane depolarization and occurs just prior to the breakdown of the nuclear membrane. Our previous studies showed [6] that intracellular pH rises from 7.37 ± 0.01 (N = 6) in the prophase-arrested oocyte to 7.82 ± 0.03 (N = 5) during the first 3 h after exposure to progesterone, consistent with utilization of H^+ ^during ATP formation via the creatine kinase reaction.

### Possible mechanism of progesterone-mediated increases in high energy phosphate compounds

The surface of the prophase amphibian oocyte [28], as well as that of most other cells [29], is studded with microscopic, flask-shaped invaginations called caveolae (~70 nm average outer diameter) that can either open for receiving and/or releasing material or close for processing and/or delivery to intracellular sites. Coinciding with the internalization of ouabain bound to the α-subunit of Na/K-ATPase, there is a net internalization of *Rana *oocyte plasma membrane and a disappearance of ouabain-sensitive K^+^-current [18]. Dersch et al. [30] report a similar progesterone-induced increase in cortical membrane trafficking in *Xenopus laevis *oocytes. By completion of the first meiotic division the cytoplasm of the progesterone-stimulated prophase oocyte becomes isopotential with the extracellular environment [31]. Xie and Askari [32] concluded from studies with cardiac cells that there are two pools of Na/K-ATPase with distinct but coupled functions. One is the classical pool in which the enzyme acts as an energy transducing ion pump and is localized in non-caveolar membranes. The other is the steroid -modulated, signal-transducing pool of the enzyme, which, through helix-helix interaction with membrane proteins called caveolins [33], is localized within the lipid rafts associated with the caveolae (reviewed in [34]). Our results suggest that progesterone acts to shift α-subunits from non-caveolar plasma membrane regions to the lipid rafts associated with caveolar membranes, followed by increased endocytosis of the caveolar vesicles. This is consistent with evidence indicating that the membrane regions containing >95% of the Na/K-ATPase are selectively internalized over a 2-3 h period prior to nuclear membrane breakdown [2].

The α-subunit of Na/K-ATPase may thus cycle between the non-caveolar regions of the plasma membrane and the caveolar membranes. Our experiments indicate that decrease in the rate constant k_f _for the PCr → ATP reaction (Figure 6) coincides with internalization of the Na-pump. The decrease in rate constant must arise from reduced ADP availability and would result in an increased cytosolic phosphoryl potential. This increased phosphoryl potential would, in turn, contribute to enhanced phosphorylation of yolk phosvitin (Figure 7, Table 1).

### Cation binding to yolk platelet phosvitin

In addition to non-heme iron [23], yolk phosvitin also contains Ca^2+^, Mg^2+^, Na^+ ^and K^+ ^[11]. Partially relaxed ^23^Na Fourier transform NMR spectra revealed the existence of at least two major intracellular compartments of NMR-visible Na^+ ^[35]. A large fraction of the *Rana *oocyte Na^+ ^was NMR-invisible and could be recovered in the yolk platelets [35]. During the first meiotic division there is a net increase in NMR-visible Na^+^; by completion of the second meiotic division (following fertilization), about 70% of the total Na^+ ^becomes NMR-visible. Thus, phosvitin not only serves as a site for energy storage, but also as a storage site for iron and other ions essential for embryonic development in ponds and streams that contain little dissolved salts and minerals.

## Conclusions

The pattern emerging from these and related studies indicates that progesterone binding to the N-terminal external loop of the α1-subunit of Na/K-ATPase initiates a cascade of events, facilitated by the internalization of Na/K-ATPase [2] and sequential changes in plasma membrane phospholipids (e.g. [36]). As shown here, progesterone binding initiates a marked rise in phosphocreatine and phosphoryl potential within the first few hours, followed by an accumulation of the highly phosphorylated protein phosvitin by the onset of second metaphase arrest. Continued binding of progesterone to the catalytic-subunit of the Na-pump is essential during the first 4-5 h [37] and leads to a net internalization of 50-60% of the total oocyte plasma membrane [18,38], which contains more than 95% of both bound progesterone and the ouabain-sensitive Na/K-ATPase [2]. We propose that progesterone binding to the catalytic-subunit of the Na/K-ATPase leads to a shift of Na/K-ATPase to lipid rafts. These rafts contain Na/K-ATPase-progesterone-caveolin-lipid microdomains [34] and undergo selective internalization prior to nuclear membrane breakdown. The resulting diversion of oxidative energy from cation regulation at the plasma membrane to storage as high energy phosphates in yolk phosvitin is essential for the subsequent fertilization and early cleavage.

## Methods

Gravid *Rana pipiens *females from the northeastern United States were purchased from Connecticut Valley Biologicals, Southhampton, MA, and maintained in hibernation at 5-8°C. Steroids were obtained from Steraloids Inc. (Newport, RI). ^32^PO_4 _(Disodium phosphate in water, 900-1100 mCi/mmol) was obtained from New England Nuclear (now Perkin Elmer Inc). Modified Ringer's solution contained 111 NaCl, 1.9 mM KCl, 1.1 mM CaCl_2_, 0.8 mM MgSO_4_, 2.3 mM NaHCO_3_, and 0.08 mM NaHPO_4_. The ionic composition of this amphibian Ringer's solution is based on that of frog plasma [39] and differs from "Barth's" or various Ringer's solutions used in *Xenopus laevis *experiments. (For example, the Ringer's solution used with *Xenopus *follicles is phosphate-free and is adjusted to pH 7.8 with Tris-HCl [27]). Progesterone and 17-β-estradiol were dissolved in 95% ethanol; 1.0 μl was added per ml of Ringer's solution with shaking for 30 sec, followed by 1:10 serial dilutions. The final ethanol content was 0.01%. Electron micrographs were prepared from oocytes as described by Weinstein et al. [18].

### Use of *Rana pipiens *oocytes

The *Rana pipiens *oocyte is particularly appropriate for studies of steroid action at the plasma membrane. Unlike *Xenopus laevis *ovaries, which contain several stages of growing oocytes [4], the mature *Rana pipiens *ovary contains 1-3 thousand fully grown oocytes in meiotic prophase arrest, that undergo synchronous meiotic divisions in response to progesterone. *Rana pipiens *oocytes grow and store yolk during the summer prior to the frog's entry into hibernation in the late fall. Oocytes and/or follicles were obtained from hibernating gravid females from December through April. Each oocyte is a giant cell, 2 - 2.3 mm in diameter. Intact plasma-vitelline membranes can be isolated and used to study ligand binding (e.g. [2]). Several hundred oocytes collected from one female are sufficient to characterize protein phosphorylation, ligand binding, sequential changes in plasma membrane potential and surface area (capacitance). Follicles or denuded oocytes can be superfused with a modified Ringer's solution in NMR tubes to monitor changes in phosphorylation state, reaction kinetics, etc. during the time course of meiosis [6].

### Induction of meiosis

Fully grown *Rana pipiens *oocytes, arrested in first meiotic prophase, are obtained by dissecting out intact ovarian follicles (oocytes enclosed in follicle cell envelopes). Oocytes are then manually stripped of their follicular envelopes and freed from adhering thecal cells [40]. The latter are herein referred to as "denuded oocytes". Denuded oocytes, follicles and ovulated eggs were handled with glass pipettes having a bore slightly larger than the oocyte, and bent about 30 degrees near the pipette tip.

**Figure 8:**
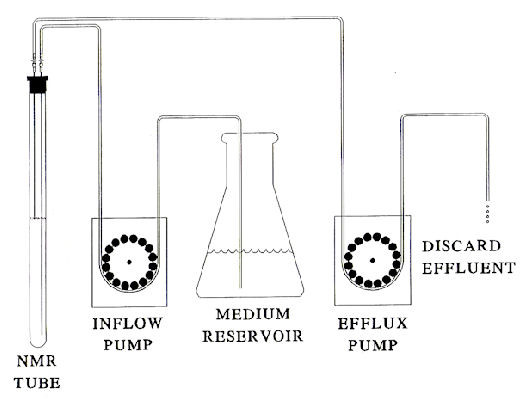
**Diagram of the apparatus used to superfuse 200-250 isolated *Rana pipiens *follicles loosely packed in a 10 mm diameter NMR tube containing modified Ringer's solution**. Measurements were made at 20-22°C in Ringers solution with or without progesterone. Using two peristaltic pumps, follicles were continuously superfused by introducing aerated Ringer's solution at the bottom of the NMR tube (1 ml/min) and drawing medium off at the top of the tube. As shown, effluent was discarded and not recycled.

Isolated follicles and/or denuded oocytes were induced to undergo meiosis by incubation in modified Ringer's solution containing progesterone at 20-22°C. Follicles were superfused in aerated NMR tubes (Figure 8) or in scintillation counting vials (20 oocytes/10 ml). The responsiveness of oocytes from each *Rana *female was determined by incubating groups of 20 oocytes or follicles in modified Ringer's solution and with or without progesterone for 12 h. Oocytes or follicles were then heat-fixed and dissected under a 10-power stereo-microscope. Completion of meiosis was determined by the disappearance of the large (0.5 mm diameter) nucleus. All data presented here were obtained from follicles/oocytes in which >95% of steroid-treated oocytes displayed nuclear membrane breakdown.

### ^32^PO_4 _Uptake and phosphorylation of total oocyte protein.

Untreated (control) and progesterone-stimulated (3.2 μM) denuded oocytes were incubated in 4.0 ml of Ringer's solution containing 80 μM [^32^PO_4_] at 20°C. Groups of six denuded oocytes were removed at the times indicated, rinsed with Ringer's solution, and homogenized in 2.0 ml 7% TCA at ice bath temperatures. The homogenate was allowed to stand for 5 min, then centrifuged and both washed precipitate and aliquots of the supernatant were counted. Uptake was corrected for estimated cell water content (50 ± 2%) and the specific activity of the medium expressed as μmoles/liter of cytoplasm.

### ^32^PO_4 _Incorporation into yolk phosvitin.

In the phosvitin labeling studies, groups of 400 follicles were labeled with 10 μCi [^32^PO_4_] in 20 ml Ringer's solution (with and without 3.2 μM progesterone) for the times indicated at 20-22°C. Follicles were also pulse labeled for 4 h, beginning 5 h after exposure to 3.2 μM progesterone. At the end of the exposure to ^32^PO_4 _containing media, the follicles were rinsed with Ringer's solution, homogenized in 5 volumes of 7% TCA at ice bath temperatures and allowed to stand at 4-5°C for 5 min. The suspension was centrifuged, the precipitate washed 2× with 3 volumes of 7% cold TCA, and extracted sequentially with 2 volumes of CHCl_3_:CH_3_OH (2:1) and CHCl_3_:CH_3_OH:HCl (2:1:0.01). Nucleic acid was extracted with 7% TCA at 90°C for 15 min, and the final precipitate treated with 0.05 N NaOH at 100°C for 10 min to recover the alkali-labile phosphate from the remaining phosphoprotein. Aliquots were counted by liquid scintillation spectrophotometry and analyzed for phosphate as described elsewhere [5]. Protein was measured by the Bicinchoninic acid method of Smith et al. [41].

Phosvitin was prepared from *Rana *ovarian follicles using a method adapted from Mano and Lipmann [42]. A more recent isolation method by McCollum et al. [43] utilized ferric ion precipitation of "specific" phosvitins. However, Taborsky has reported [44] that ferric ions caused dephosphorylation of egg yolk proteins at alkaline pH. It should be noted that non-heme iron is recovered with purified phosvitin [23] and is present as a contaminant in laboratory grade salts used in protein isolation. To reduce possible protein dephosphorylation during purifcation, 10 mM EDTA was added to all solutions used to isolate oocyte phosvitin.

The highest yields of phosvitin were obtained from *Rana *follicles as follows: 400 follicles (~900 mg wet weight) were homogenized in 1/2 volume of a solution of 0.15 M KCl, 0.025 M NaHCO_3_, 0.02 M NaF and 0.01 M EDTA (disodium salt) in a Potter-Elvehjem homogenizer at ice-bath temperatures. The homogenate was diluted with 1.5 volumes of the extraction solution and centrifuged at 13,000 × g for 60 min. 1/10 volume of 1M barium acetate was added to the supernatant and the pH adjusted to about 7.0 with dilute NH_4_OH. After standing for 30 min at ice-bath temperatures, the precipitate was collected by centrifugation and resuspended in 0.5 volumes of 0.2 M ammonium sulfate containing 0.05 M Tris buffer, pH 7.6. The resulting barium sulfate ppt was removed by centrifugation, and the opalescent solution dialyzed against distilled water for 48 h at 5°C. In an ice-bath, 1.5 volumes of 95% ethanol were added to precipitate the protein, which was washed with CHCl_3_:CH_3_OH (2:1), then dried and ground in a mortar. The powder contained 0.75 ± 0.04% alkali-labile phosphorous (N = 3), and represented 59 ± 4% (N = 3) recovery.

### Nuclear magnetic resonance procedures

Follicular oocytes were prepared as described above and continuously superfused (1 ml/min) with aerated Ringer's solution at 20°C in a 10 mm NMR tube as illustrated in Figure 8. ^31^P NMR experiments were carried out using a Varian VXR 500 spectrometer operating at 202 MHz with a spectral width of 20 KHz. Assignment of ^31^P-peaks were determined as described by Nuccitelli et al. [45]. Saturation transfer experiments were performed by application of a low power radio frequency (RF) pulse for a time >3T_1 _to either the γATP or PCr resonance [12,46]. Control spectra were obtained by positioning the saturating pulse off-resonance on the other side of PCr or γATP, respectively, at the same distance from the observed resonance. Both control and appropriately saturated spectra were accumulated in alternate blocks of 100 scans for a total of 1000 scans each. Pseudo first order rate constants were calculated from the extent of the saturation transfer effect measured in the difference spectrum. NMR measures the pseudo first order rate constant k_f _= k_1_[ADP], where k_1 _is the true second order rate constant for the creatine kinase reaction. Quantitation of phosphometabolites was accomplished from ^31^P NMR of oocytes by comparing the areas of the various fully relaxed resonances with that of a standard phosphate sample, taking into account the fraction of the NMR-sensitive window occupied by intracellular H_2_0 [6].

## Conflict of interest

The authors declare that they have no competing interests.

## Authors' contributions

The approach was conceived of, and the experiments were carried out, by all four authors. GAM wrote the first draft of the manuscript and it was edited by all authors. All authors read and approved the final manuscript.
